# Targeted next-generation sequencing of malignant peripheral nerve sheath tumor of the pterygopalatine fossa with intracranial metastatic recurrence

**DOI:** 10.1097/MD.0000000000009636

**Published:** 2018-01-26

**Authors:** Xinjie Bao, Xiangyi Kong, Chengxian Yang, Huanwen Wu, Wenbin Ma, Renzhi Wang

**Affiliations:** a Department of Neurosurgery; b Department of Pathology, Peking Union Medical College Hospital, Chinese Academy of Medical Sciences & Peking Union Medical College, Beijing, China.

**Keywords:** DNA sequencing, intracranial, malignant peripheral nerve sheath tumor, recurrent

## Abstract

Malignant peripheral nerve sheath tumor (MPNST) is an uncommon neoplasm that rarely involves the head and neck region. Intracranial MPNSTs unrelated to cranial nerves are highly malignant tumors with poor overall survival, probably because of infiltrating growth into surrounding brain tissue. The pathogenesis of MPNST remains unclear. There are no conclusive explanations for the mechanisms underlying the initiation, progression, and metastasis of MPNST. In this paper, we describe a case of MPNST in the pterygopalatine fossa with intracranial metastatic recurrence and review related literatures. Meanwhile, targeted next-generation sequencing (NGS) revealed the presence of both a beta-catenin (CTNNB1) missense mutation p.Ser33Phe and a mediator complex subunit 12 (MED12) frameshift mutation p.Tyr1278fs in the recurrent intracranial tumor. Therapies that target CTNNB1 mutation, MED12 mutation, CTNNB1 activation, or Wnt pathway activation are worth future studying.

## 1 Introduction

Malignant peripheral nerve sheath tumor (MPNST) was previously known as malignant schwannoma, neurogenic sarcoma, malignant neurilemmoma, and neurofibrosarcoma. MPNST is a rare neoplasm that originates in nerve sheath cells, including Schwann cells and neural and perineural fibroblasts. It accounts for about 5% to 10% of all soft-tissue sarcomas, and its prevalence among the general population is around 1 per 100,000. The presence of an MPNST in the head and neck and involvement of the central nervous system are very uncommon. The histogenesis of MPNST remains unclear. There is a dismal prognosis for this disease, for which surgical intervention remains the mainstay for treatment. The value of chemotherapy remains questionable. In this paper, we report a case of a pterygopalatine fossa MPNST with metastasis into the intracranial compartment. We used targeted next-generation sequencing (NGS) technology to sequence a panel of genes in an attempt to discover targetable genetic changes and to decipher the pathogenesis of MPNST.

## 2 Case presentation

### 2.1 The first 2 operations

A 24-year-old Chinese student presented with a month's history of diplopia. She had a history of what was reported to be a benign tumor associated with her mandible. This was operated on in China with a pathological diagnosis of “myofibroma.” The family was told that this was benign but that postoperative radiotherapy was recommended to reduce recurrence although they were informed that the whole tumor had been removed. Since that time, she had been well with no other medical problems. Investigations with computed tomography (CT) and magnetic resonance imaging (MRI) showed a multilobulated patchily enhancing tumor in the middle cranial fossa abutting on the cavernous sinus with expansion up to within a millimeter of the middle cerebral artery and occupying most of the anteromedial artery of that fossa. The tumor extended down through the foramen rotundum and through the inferior orbital fissure into the infratemporal fossa and lobulated mass were seen within the temporalis muscle extending down to the branches of the trigeminal nerve. Risks associated with resection of this lesion were fully discussed including the fact that she would lose facial sensation almost definitely as well as eye movement of the left eye and she would develop a ptosis due to involvement of the 3rd, 4th, 5th, and 6th nerves on the left.

Under general anesthesia with endotracheal intubation, the head, neck, and face were prepped and draped in a sterile fashion. A curvilinear incision was made from the anterior hairline passing posteriorly and then laterally down toward the temporal region and into the preauricular crease. A flap was raised in the galeal layer and the frontal branch of the facial nerve preserved in the fat pad as the entire flap was elevated forward to expose the frontal bone, temporal bone, zygoma, and orbit. Temporalis was incised and the anterior portion reflected inferiorly into the fossa. There was significant tumor noted in the temporal fossa and within the temporalis muscle, some of which was excised. A zygomatic osteotomy was made with a gigli's saw, fontal cuts were also made and a large bone flap incised and then elevated and the bone put aside. As the bone flap was elevated it was obvious tumor was extending into the pterygo-maxillary fissure and involving the pterygoid muscles as they came off the pterygoid plate. This area was completely excised. The intro-orbital and intro-cranial portions of this resection and the closure were performed smoothly. The emporalis muscle was finally completely resected as it was grossly involved with tumor nodules. The surgery went well. One week after the surgery, the patient was discharged from hospital. Then she underwent radiotherapy and no obvious discomfort and correlative complication were found.

### 2.2 Pathological examination

*Gross description*: The specimen consists of an irregular piece of rubbery tan to brown tumor 35 × 30 × 15 mm. *Microscopy*: Sections of this tissue and tissue show a variably cellular tumor composed of lump moderately pleomorphic spindled cells within a variable fibrous stroma. Areas of vague nuclear palisading are present and the tumor contains prominent areas of hyalinized cords and nodules characteristic of MPNST. The nodules, which are present widely through the tumor, are composed of circular arrays of tumor cells with the long axes of the nuclei arranged in a spoke-like pattern around the circumference from which fibrils of collagen extend to the center creating pseudorosette structures. There structures are described as distinctive but uncommon features of MPNST. The tumor has variable cellularity, with the more cellular areas having a mitotic rate of 5 mitoses per 10 high power fields. There is one area of probable coagulative necrosis, however this is near a margin and necrosis is otherwise not identified. There is no evidence of skeletal muscle or glandular differentiation within the tumor. The diagnosis of MPNST is also supported by the immunohistochemical profile of the tumor-moderate diffuse positivity for S100 and vimentin, and no staining for CD34, CD10, Desmin, MSA, SMA, AE1/3, EMA, and Caldesmin.

The grading of the tumor is slightly problematic as the relatively low mitotic rate and strong S100 positivity are most consistent with a WHO grade III tumor; however, the small focus of possible necrosis could elevate it to WHO IV. Overall the features are interpreted as being most consistent with WHO grade III. Considering the patient's youthful age and the rarity of MPNST of the cranial nerves (the presumed site of origin of this tumor) neurofibromatosis type 1 should be excluded in this patient. So, the final pathological diagnosis was MPNST WHO III.

### 2.3 The third operation

**Figure 1:**
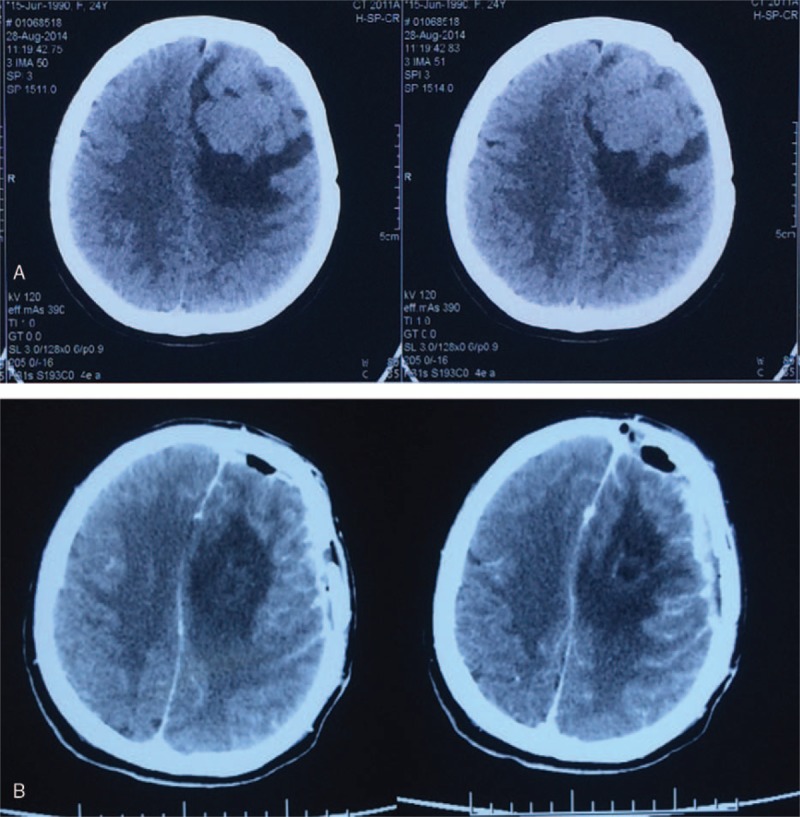
Preoperative cerebral CT showed a new local hypermetabolic foci in the left frontal lobe (A). Postoperative cerebral CT showed the tumor was completely resected (B). CT = computed tomography.

**Figure 2:**
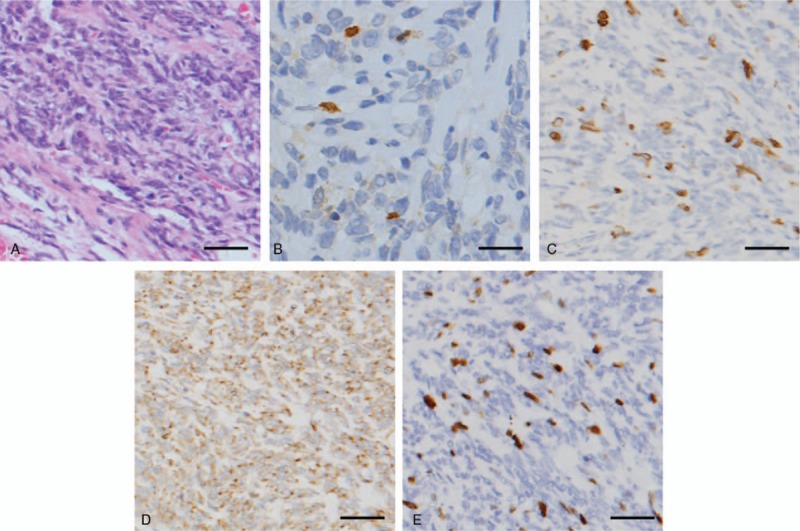
The postoperative pathology examination results were consistent with the diagnosis of MPNST. H&E × 100 (A) S100 × 100 (B) NF × 100 (C) NSE × 100 (D); Ki-67 × 100 (E). Scale bar: 20 μm. MPNST = malignant peripheral nerve sheath tumor.

Four years later, the patient complained of dizziness, dizzy, nausea, and vomiting sometimes. She came to our hospital for postoperative re-examination. Cerebral CT, MRI, and positron emission tomography-CT showed that there was a new local hypermetabolic foci in her left frontal lobe, which was very likely to be an ectopic recurrence (Fig. 1A). Around the lesion was slightly edema belt. Interiorly behind her left orbit in the frontal lobe was a patchy mass with soft tissue density with increased metabolism, which might be the residual tumor or a recurrence. Metabolic reduction of the left cerebral cortex, basal ganglia suggested a possible secondary function change. Her left eyeball could not move abductively very well. The left eye could only see fingers moving within 30 cm. the pupillary light reflex of her left eye is insensitive. Physical examination was normal, with no stigmata of NF-1. A chest x-ray, abdominal ultrasonography, and bone scintigraphy revealed no signs of metastatic disease. After multidisciplinary discussion, a third craniotomy and intracranial lesion resection was performed. During the surgery, the tumor was gray-red and rubbery, with abundant blood supply. The tumor was relatively circumscribed with a surrounding edema belt. The tumor underneath the galea aponeurotica adjacent to the cerebral falx in the left frontal lobe was completely resected (Fig. 1B). The mass was about 6 cm × 4 cm × 4 cm. The postoperative pathology examination (Fig. 2) showed that most of the cells were malignant spindle cells. The immunohistochemical results: CD34(−), Desmin (−), GFAP(−), Ki-67% (index 20%), S-100(±), SMA(−), Bcl-2(+), Nestin(+), AE1/AE3(−), CD117(−), CD99(+), EMA(−), HMB45(−), NSE(+), WT-1(−), Fli-1(+). Taking together, the pathology results were consistent with the diagnosis of MPNST.

## 3 Materials and methods

### 3.1 Study samples

Tumor DNA was extracted from formalin-fixed paraffin-embedded (FFPE) samples and fresh frozen samples from the third operation using the QIAamp DNA FFPE Tissue Kit and the DNEasy Blood and Tissue Extraction Kit (Qiagen, Hilden, Germany), respectively, based on the manufacturer instructions. All FFPE and fresh frozen tissue samples underwent H&E staining and were reviewed by a pathologist to ensure > 70% tumor content. The DNA purity and concentration were assessed using a NanoDrop2000 spectrophotometer; the DNA qualities were assessed by agarose-gel electrophoresis. The normal sample was the patient's whole blood.

### 3.2 Targeted next-generation sequencing

Library construction was performed as previously described using 1 μg of DNA sheared by an ultrasonoscope to generate fragments with a peak length of 250 bps, followed by end repair, a tailing and ligation to the Illumina-indexed adapters according to the standard library construction protocol. Target enrichment was conducted on a custom sequence captureprobe (Nimblegen), which targeted 7708 exons of 508 cancer-related genes and 78 introns from 19 genes recurrently rearranged in solid tumors, representing approximately 1.7 Mb of the human genome in total. Sequencing was performed with 2 × 101-bp paired-end reads and an 8-bp index read on an Illumina Hiseq 2500 platform (Illumina, San Diego) using the manufacturer's protocols. Genotype calling of multiallelic substitutions and indels was performed on each individual sample using Torrent Variant Caller (TVC) version 4.4.3. Sequence variants were annotated using wANNOVAR (http://wannovar.usc.edu/). Sequence data were visualized using the Integrative Genome viewer (http://www.broadinstitute.org/igv/).

### 3.3 Mutation confirmation

**Figure 3:**
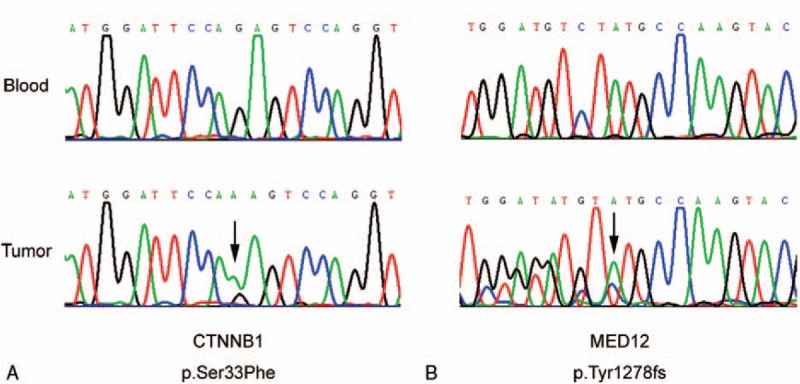
Sanger sequencing reveals that the CTNNB1 mutation is a missense one, p.Ser33Phe (A) and the MED12 mutation is a frameshift one, p.Tyr1278fs (B). CTNNB1 = beta-catenin, MED12 = mediator complex subunit 12.

**Figure 4:**
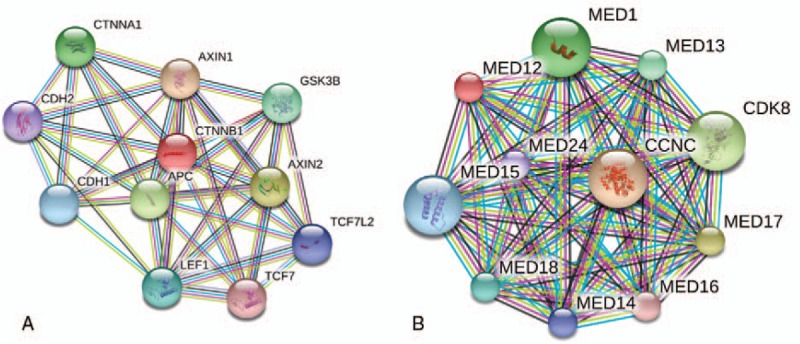
The protein–protein interaction (PPI) network and the screen results of gene CTNNB1 and MED12 based on the information in the STRING database. CTNNB1 = beta-catenin, MED12 = mediator complex subunit 12, PPI = protein–protein interaction.

In this study, the mutations were detected for cancer-related genes, including proto oncogene and tumor suppressor gene, drug target gene, high frequency mutation gene of tumor, genetic predisposing gene of tumor, and important information of 12 tumor signal pathways. Among them, 195 genes were screened for mutations, insertions, and deletions (Table 1); 24 genes were screened for gene rearrangement (Table 2); and 69 genes were for copy number variations (CNV) (Table 3). Upon the DNA sequencing, results showed that there were mutations with the tumor gene beta-catenin (CTNNB1) and mediator complex subunit 12 (MED12). The CTNNB1 mutation is a missense one, p.Ser33Phe (Fig. 3A) and the MED12 mutation is a frameshift one, p.Tyr1278fs (Fig. 3B). The overview of the sequencing data (tumor molecular pathological analysis) is shown in Table 4. To determine a protein–protein interaction (PPI) network, based on the information in the STRING database, the 2 genes were screened. The related hub genes included CDH2, CTNNA1, AXIN1, GSK3B, TCF7L2, LEF1, and APC et al for CTNNB1, and CDK8, CCNC, MED1, MED13, and MED 24, etc., for MED12 (Fig. 4).

**Table 1:**
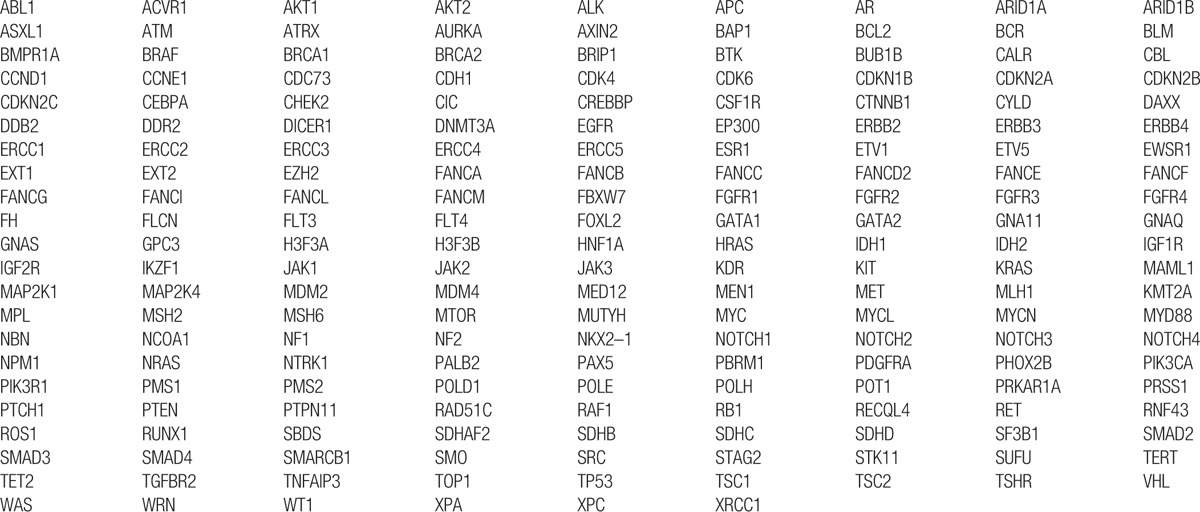
A total of 195 genes were screened for mutations, insertions, and deletions.

**Table 2:**
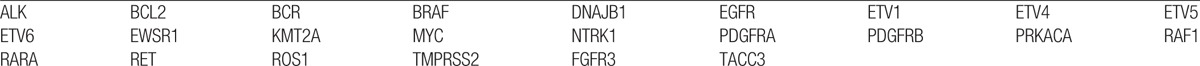
Around 24 genes were screened for gene rearrangement.

**Table 3:**
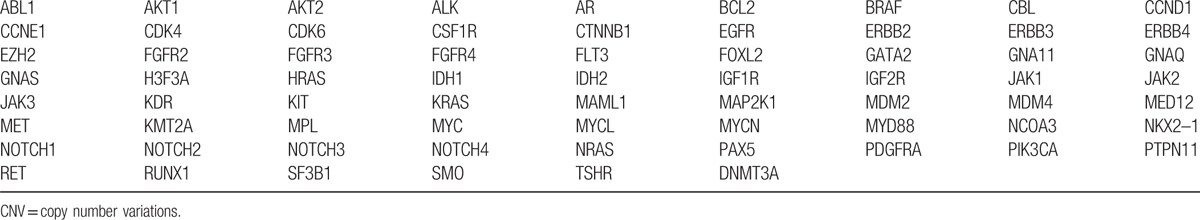
Around 69 genes were screened for copy number variations (CNV).

**Table 4:**
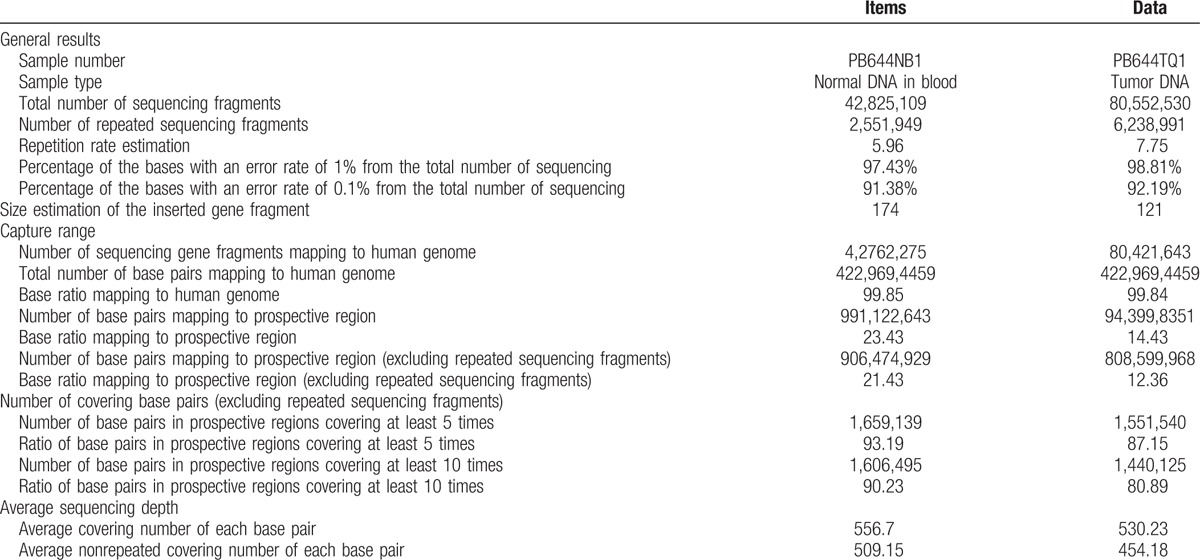
The overview of the sequencing data (tumor molecular pathological analysis).

## 4 Discussion

### 4.1 MPNSTs

**Figure 5:**
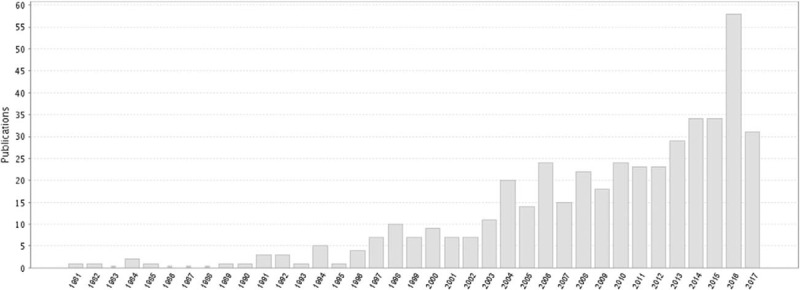
The studies of MPNST have shown a tendency to increase by year. MPNST = malignant peripheral nerve sheath tumor.

**Figure 6:**
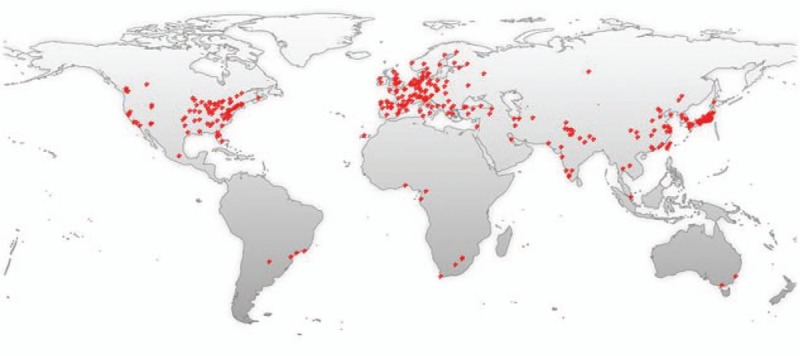
The publications are mainly from East Asia, North America, and Europe.

MPNST are an uncommon but devastating tumor of peripheral nerve, representing only about 10% of tumors encountered by a peripheral nerve surgeon. The incidence of MPNST in general population is 0.001%.^[1]^ The studies of MPNST have shown a tendency to increase by year (Fig. 5). The publications are mainly from East Asia, North America, and Europe (Fig. 6). MPNST is mostly found on trunk and extremities, and less often on the neck and head. Intracranial MPNSTs arising in cranial nerves are very rare, and the literatures are limited to small case series or isolated cases.^[2]^ Intracranial MPNSTs unrelated to cranial nerves are even rarer. According to van den Munckhof et al's review, only 22% of the cases arose in patients with NF-1, compared with 650% of extracranial MPNSTs.^[3]^ The clinical picture is characterized by rapid change, whether in pain, size of tumor mass, or progression of neurologic deficit, especially when occurring in a pre-existing peripheral nerve tumor. In particular, change in tumor size is most predictive of malignancy. Major nerve trunks (e.g., the brachial plexus) are frequent sites of involvement, but a nerve of origin is often not evident.

The diagnosis of MPNST is difficult. It is based on a combination of gross, histopathologic, and immunohistochemical findings.^[4]^ The histological findings are quite variable. Based on the descriptions by Valdueza of the histopathologic features of intracranial MPNSTs, the most reliable criteria for malignancy are endothelial proliferations, necrosis, increased mitotic rate, nuclear atypia, and high cellularity; and the clearly malignant areas may be present only in isolated areas.^[5]^ Because the histology is not very specific, immunohistochemistry plays a major role in the diagnosis. The most important immunohistochemical finding is the presence of focal positivity for S-100 protein, which is seen in as many as 75% of cases.^[6]^ S-100 positive cells are not observed in other spindle-cell sarcomas. Additionally, some MPNST cases stain positively for neuron-specific enolase. Staining for cytokeratins and EMA reactivity is usually negative.

Treatment of these aggressive neoplasms is not standardized. The most widely accepted treatment is radical excision with free margins when possible.^[7]^ This is sometimes not feasible because of the size or location of the tumor, especially in head and neck cases, which may explain the poor prognosis among these patients. The role of adjuvant therapy is not clear.^[7]^ Neither radiotherapy nor chemotherapy has consistently been shown to increase survival rates, although postoperative radiotherapy is widely used to control local recurrences. Chemotherapy is usually reserved for patients who experience a systemic relapse, and multiple regimens have been used. Generally, the prognosis of MPNST is not good. Poorly prognostic signs include tumors > 5 cm in size, inabilities to achieve tumor-free margins, distant metastases at the time of diagnosis, older age, association with neurofibromatosis type 1, and higher tumor WHO grading.^[8,9]^

### 4.2 DNA sequencing

Because the central nervous system is generally devoid of peripheral nerve sheath cells, the pathogenesis of intracranial MPNSTs unrelated to cranial nerves has been explained by several theories. Thus, besides presenting this rarity, another objective of our study was to gain insight into the pathogenesis of disease progression of the MPNST. The mechanism that contributes to the disease initiation, progression, and metastasis remains unclear. The present study proposed a mechanism for disease progression in which combined CTNNB1 missense mutation p.Ser33Phe and MED12 frameshift mutation p.Tyr1278fs promote tumor progression in MPNST.

#### 4.2.1 CTNNB1

CTNNB1-encoded protein is part of a complex of proteins that constitute adherens junctions. Through controlling cell adhesion and cell growth, adherens junctions play key roles in the maintenance and creations of epithelia cells. The CTNNB1 protein could bind to APC gene product. The mutation in APC may lead to a series of cancers such as ovarian cancers, medulloblastomas, pilomatrixomas, and colorectal cancers. There have been 3 transcription variants encoding the same protein for the gene. CTNNB1 p.Ser33Phe is a commonly reported missense mutation located within exon 3 which encodes the phosphorylation domain of CTNNB1.^[10]^ This alteration is known to drive nuclear localizations and disrupt phosphorylations.^[11]^ CTNNB1 mutations have not been observed in any of malignant peripheral nerve sheath tumor samples sequenced in COSMIC (COSMIC, November 2015).

CTNNB1-encoded beta-catenin is a downstream-effector in WNT signalling pathway, functioning as one of the key signals for proliferations and differentiations.^[10,12]^ Altered expression of CTNNB1 can lead to abnormal signalling in various diseases, including induction of malignant pathways in cancer; it can act as an oncogene and modulate gene transcriptions to initiate cancers’ occurrences, progressions, survivals, and relapses.^[13]^ Missense mutations in the amino-terminal region of CTNNB1 affecting codons 32 to 45 have been reported in various mesenchymal and epithelial neoplasms, including colorectal carcinoma, hepatocellular carcinoma, and desmoid-type fibromatosis, and their function in maintaining aberrant activation of the Wnt signalling pathway has been well demonstrated.^[14–16]^ CTNNB1 mutations, particularly those in exon 3, are related to Wnt-pathway's activation and increased CTNNB1 protein stability.^[15,17]^ At the present time, there are no approved therapies that target CTNNB1 mutation, CTNNB1 activation, or Wnt pathway activation. Wnt pathway inhibitors, such as PRI-724, are under investigation in early clinical trials for solid tumors.^[18,19]^ The inhibition of Wnt/CTNNB1 signaling is an area of active preclinical and early clinical research.^[20,21]^ In the case of this uncharacterized variant, the relevance of any available therapeutic approaches is unknown.

Early results from a Phase 1 study of the Wnt pathway inhibitor OMP-54F28 (FZD8-Fc) in solid tumors has reported that OMP-54F28 was well tolerated through 10 mg/kg, and stable disease was achieved in 18% (3/17) of patients for 2 to 3 months.^[22]^ A preclinical study reported that sulindac benzylamine resulted in reduced nuclear beta catenin levels, and inhibited the growth and induced apoptosis of colon tumor cells.^[20]^ Another preclinical study showed that treatment of colorectal cancer cells and mouse xenografts with destruxin B, a Wnt pathway inhibitor, resulted in cell cycle arrest and inhibition of tumorigenesis, respectively.^[21]^ Overexpression of the Wnt antagonist Dickkopf-1 was shown preclinically to reduce proliferation, migration, and invasion of colon cancer cells, and to suppress tumor growth in xenograft models of colon cancer; Dickkopf-1 expression was found to inhibit E-cadherin and vimentin expression, and change the expression pattern of CTNNB1 from nuclear to mainly cytoplasmic.^[23]^

#### 4.2.2 MED12

MED12 protein is essential for activating CDK8 kinase. Defects in this gene lead to X-linked Opitz-Kaveggia syndrome, also known as and Lujan–Fryns syndrome and FG-syndrome. p.Tyr1278fs is a frameshift mutation which may result in truncated Med12 protein. Therefore, although p.Tyr1278fs has not been reported (COSMIC, November 2015) or functionally characterized, it is predicted to be an inactivating mutation. MED12 mutations have not been observed in any of malignant peripheral nerve sheath tumor samples sequenced in COSMIC (COSMIC, November 2015).

Mutation and loss of MED12 have been reported in several types of cancer; it has been suggested that inactivation of Med12 may promote cancer through the dysregulation of several signalling pathways, including TGF-beta, mitogen-activated protein kinase kinase (MEK), extracellular-regulated protein kinases, and hormone receptors. Currently no therapies directly targeting MED12 alterations have been approved or under clinical investigations. A preclinical study has reported that Med12 is a component of the Wnt/CTNNB1 pathway that is required for the transcription activation.^[24]^ MED12 activating mutations may therefore predict sensitivity to therapies targeting the Wnt/CTNNB1 pathway. Wnt pathway inhibitors are currently being studied preclinically and in early clinical trials for solid tumors.^[18,25,26]^ A preclinical study has indicated that Med12 can bind and negatively regulate Tgfbr2, and that loss of Med12 function is associated with increased TGF-beta signalling and resistance to anti-cancer therapeutics, including inhibitors of anaplastic lymphoma kinase, EGFR, MEK, and B-Raf proto-oncogene, serine/threonine kinase.^[27]^ Loss of Med12 function may therefore predict sensitivity to therapies targeting TGFBR2 or Tgfbr2, such as Lucanix and IMC-TR1, which have been in development and are in clinical trials in certain tumor types or in solid tumors.^[28,29]^ Small-molecule inhibitors of the TGF-beta pathway are also in development, but because TGF-beta can exert both tumor suppressor as well as pro-tumor effects, the development of therapies aimed at this pathway must be carefully considered for each patient.^[28]^ Inactivation of MED12 was identified as a cause of crizotinib resistance in a nonsmall cell lung carcinoma cell line. Inhibition of the TGF-beta pathway was able to restore sensitivity of nonsmall cell lung carcinoma cells to crizotinib and gefitinib.^[27]^

A Phase 1/2 trial of the TGFB2-specific antisense oligonucleotide trabedersen in patients with various solid tumors, including colorectal carcinoma, melanoma or pancreatic cancer, has reported that the treatment was safe and well tolerated, though further efficacy studies are pending. Early results from a Phase 1 study of the Wnt pathway inhibitor OMP-54F28 (FZD8-Fc) in solid tumors has reported that OMP-54F28 was well tolerated through 10 mg/kg, and stable disease was achieved in 18% (3/17) of patients for 2 to 3 months. A Phase 1 trial of PRI-724 in solid tumors has reported that the therapy has an acceptable toxicity profile. A preclinical study has reported that the dual TGFβR kinase inhibitor LY2109761 was associated with reduced cell migration and the induction of an epithelioid phenotype in cancer cell lines.^[28]^ Another preclinical study has reported the development of an anti-TGFbeta RII antibody (IMC-TR1) that was associated with increased apoptosis and necrosis, and decreased primary tumor growth, decreased metastasis in tumor models.

### 4.3 Limitations

One major limitation of our study was that the number of sequenced genes was limited. The targeted NGS only included the predefined exonic regions of 288 genes. Therefore, epigenetic changes, genomic rearrangement, and genes outside of that panel could not be detected. As the incidence of MPNST is so low, not enough specimen are available for the sequencing. More researches with larger sample size are needed in the future.

## 5 Conclusion

In conclusion, the present study provided evidence that the initiation and rapid progression of MPNST are likely to result from the concurrent mutations in CTNNB1 and MED12 genes. Therapies that target CTNNB1 mutation, MED12 mutation, CTNNB1 activation, or Wnt pathway activation are worth studying.

## References

[1] Kim DH Murovic JA Tiel RL A series of 397 peripheral neural sheath tumors: 30-year experience at Louisiana State University Health Sciences Center. J Neurosurg 2005;102:246–55. 15739552 10.3171/jns.2005.102.2.0246

[2] Patel TD Shaigany K Fang CH Comparative analysis of head and neck and non-head and neck malignant peripheral nerve sheath tumors. Otolaryngol Head Neck Surg 2016;154:113–20. 26408559 10.1177/0194599815606700

[3] van den Munckhof P Germans MR Schouten-van Meeteren AY Recurring intracranial malignant peripheral nerve sheath tumor: case report and systematic review of the literature. Neurosurgery 2011;68:E1152–8. discussion E1159. 21242836 10.1227/NEU.0b013e31820a1599

[4] Bhattacharyya AK Perrin R Guha A Peripheral nerve tumors: management strategies and molecular insights. J Neurooncol 2004;69:335–49. 15527099 10.1023/b:neon.0000041891.39474.cb

[5] Valdueza JM Hagel C Westphal M Primary spinal malignant schwannoma: clinical, histological and cytogenetic findings. Neurosurg Rev 1991;14:283–91. 1791943 10.1007/BF00383263

[6] Tamarit M Navarro R Alcazar L Malignant peripheral nerve sheath tumor of the infratemporal fossa with intracranial extension. Ear Nose Throat J 2010;89:596–9. 21174279

[7] Bradford D Kim A Current treatment options for malignant peripheral nerve sheath tumors. Curr Treat Options Oncol 2015;16:328. 25777573 10.1007/s11864-015-0328-6

[8] Amirian ES Goodman JC New P Pediatric and adult malignant peripheral nerve sheath tumors: an analysis of data from the surveillance, epidemiology, and end results program. J Neurooncol 2014;116:609–16. 24390465 10.1007/s11060-013-1345-6

[9] Kolberg M Holand M Agesen TH Survival meta-analyses for >1800 malignant peripheral nerve sheath tumor patients with and without neurofibromatosis type 1. Neuro Oncol 2013;15:135–47. 23161774 10.1093/neuonc/nos287 PMC3548581

[10] Lazar AJ Calonje E Grayson W Pilomatrix carcinomas contain mutations in CTNNB1, the gene encoding beta-catenin. J Cutan Pathol 2005;32:148–57. 15606674 10.1111/j.0303-6987.2005.00267.x

[11] Polakis P Hart M Rubinfeld B Defects in the regulation of beta-catenin in colorectal cancer. Adv Exp Med Biol 1999;470:23–32. 10709671 10.1007/978-1-4615-4149-3_3

[12] van Es JH Barker N Clevers H You Wnt some, you lose some: oncogenes in the Wnt signaling pathway. Curr Opin Genet Dev 2003;13:28–33. 12573432 10.1016/s0959-437x(02)00012-6

[13] Thakur R Mishra DP Pharmacological modulation of beta-catenin and its applications in cancer therapy. J Cell Mol Med 2013;17:449–56. 23490077 10.1111/jcmm.12033 PMC3822645

[14] Stamos JL Weis WI The beta-catenin destruction complex. Cold Spring Harb Perspect Biol 2013;5:a007898. 23169527 10.1101/cshperspect.a007898 PMC3579403

[15] Polakis P The oncogenic activation of beta-catenin. Curr Opin Genet Dev 1999;9:15–21. 10072352 10.1016/s0959-437x(99)80003-3

[16] MacDonald BT Tamai K He X Wnt/beta-catenin signaling: components, mechanisms, and diseases. Dev Cell 2009;17:9–26. 19619488 10.1016/j.devcel.2009.06.016 PMC2861485

[17] Notani D Gottimukkala KP Jayani RS Global regulator SATB1 recruits beta-catenin and regulates T(H)2 differentiation in Wnt-dependent manner. PLoS Biol 2010;8:e1000296. 20126258 10.1371/journal.pbio.1000296 PMC2811152

[18] Kogan Y Halevi-Tobias KE Hochman G A new validated mathematical model of the Wnt signalling pathway predicts effective combinational therapy by sFRP and Dkk. Biochem J 2012;444:115–25. 22356261 10.1042/BJ20111887

[19] Lachenmayer A Alsinet C Savic R Wnt-pathway activation in two molecular classes of hepatocellular carcinoma and experimental modulation by sorafenib. Clin Cancer Res 2012;18:4997–5007. 22811581 10.1158/1078-0432.CCR-11-2322 PMC3446854

[20] Whitt JD Li N Tinsley HN A novel sulindac derivative that potently suppresses colon tumor cell growth by inhibiting cGMP phosphodiesterase and beta-catenin transcriptional activity. Cancer Prev Res (Phila) 2012;5:822–33. 22556201 10.1158/1940-6207.CAPR-11-0559 PMC3546530

[21] Yeh CT Rao YK Ye M Preclinical evaluation of destruxin B as a novel Wnt signaling target suppressing proliferation and metastasis of colorectal cancer using non-invasive bioluminescence imaging. Toxicol Appl Pharmacol 2012;261:31–41. 22465936 10.1016/j.taap.2012.03.007

[22] Le PN McDermott JD Jimeno A Targeting the Wnt pathway in human cancers: therapeutic targeting with a focus on OMP-54F28. Pharmacol Ther 2015;146:1–1. 25172549 10.1016/j.pharmthera.2014.08.005 PMC4304994

[23] Qi L Sun B Liu Z Dickkopf-1 inhibits epithelial–mesenchymal transition of colon cancer cells and contributes to colon cancer suppression. Cancer Sci 2012;103:828–35. 22321022 10.1111/j.1349-7006.2012.02222.x PMC7659378

[24] Kim S Xu X Hecht A Mediator is a transducer of Wnt/beta-catenin signaling. J Biol Chem 2006;281:14066–75. 16565090 10.1074/jbc.M602696200

[25] Tanwar PS Zhang L Kaneko-Tarui T Mammalian target of rapamycin is a therapeutic target for murine ovarian endometrioid adenocarcinomas with dysregulated Wnt/beta-catenin and PTEN. PLoS One 2011;6:e20715. 21695255 10.1371/journal.pone.0020715 PMC3111436

[26] Zhu J Zhang S Gu LY Epigenetic silencing of DKK2 and Wnt signal pathway components in human ovarian carcinoma. Carcinogenesis 2012;33:2334–43. 22964660 10.1093/carcin/bgs278

[27] Huang SD Holzel M Knijnenburg T MED12 controls the response to multiple cancer drugs through regulation of TGF-beta receptor signaling. Cell 2012;151:937–50. 23178117 10.1016/j.cell.2012.10.035 PMC3672971

[28] Bharathy S Xie W Yingling JM Cancer-associated transforming growth factor beta type II receptor gene mutant causes activation of bone morphogenic protein-Smads and invasive phenotype. Cancer Research 2008;68:1656–66. 18339844 10.1158/0008-5472.CAN-07-5089 PMC2667898

[29] Akhurst RJ Hata A Targeting the TGF beta signalling pathway in disease. Nat Rev Drug Discov 2012;11:790–811. 23000686 10.1038/nrd3810 PMC3520610

